# Ophthalmic Myology

**Published:** 1904-03

**Authors:** 


Ophthalmic Myology. A Systematic Treatise on the Ocular Muscles. By G. C. Savage, M. D., Professor of Ophthalmology in the Medical Department of the Vanderbilt University; author of "New Truths in Ophthalmology;" ex-President of the Nashville Academy of Medicine; ex-President of the Tennessee State Medical Society. Sixty-one illustrative cuts and six plates. Price, $4.00. 1903.

This work has been carefully prepared, and in some respects it diverges from heretofore beaten paths. It is to an author's credit sometimes that the beaten path has not been followed. Too many books are only a change of verbiage, and for the good of science had better remained unwritten. Savage's work should interest any one at all familiar with ophthalmology. T. J. B.

